# A guide for developing comprehensive systems biology maps of disease mechanisms: planning, construction and maintenance

**DOI:** 10.3389/fbinf.2023.1197310

**Published:** 2023-06-22

**Authors:** Alexander Mazein, Marcio Luis Acencio, Irina Balaur, Adrien Rougny, Danielle Welter, Anna Niarakis, Diana Ramirez Ardila, Ugur Dogrusoz, Piotr Gawron, Venkata Satagopam, Wei Gu, Andreas Kremer, Reinhard Schneider, Marek Ostaszewski

**Affiliations:** ^1^ Luxembourg Centre for Systems Biomedicine (LCSB), University of Luxembourg, Esch-sur-Alzette, Luxembourg; ^2^ Independent Researcher, Massy, France; ^3^ Université Paris-Saclay, Laboratoire Européen de Recherche Pour la Polyarthrite Rhumatoïde–Genhotel, University Evry, Evry, France; ^4^ Lifeware Group, Inria Saclay-Ile de France, Palaiseau, France; ^5^ ITTM Information Technology for Translational Medicine, Esch-sur-Alzette, Luxemburg; ^6^ Computer Engineering Department, Bilkent University, Ankara, Türkiye; ^7^ ELIXIR Luxembourg, Belvaux, Luxembourg

**Keywords:** disease mechanisms, curation, pathway biology, systems biology, translational research

## Abstract

As a conceptual model of disease mechanisms, a disease map integrates available knowledge and is applied for data interpretation, predictions and hypothesis generation. It is possible to model disease mechanisms on different levels of granularity and adjust the approach to the goals of a particular project. This rich environment together with requirements for high-quality network reconstruction makes it challenging for new curators and groups to be quickly introduced to the development methods. In this review, we offer a step-by-step guide for developing a disease map within its mainstream pipeline that involves using the CellDesigner tool for creating and editing diagrams and the MINERVA Platform for online visualisation and exploration. We also describe how the Neo4j graph database environment can be used for managing and querying efficiently such a resource. For assessing the interoperability and reproducibility we apply FAIR principles.

## 1 Introduction

Disease maps have been introduced in systems biomedicine as conceptual models of disease mechanisms (Mazein et al., 2018; Ostaszewski et al., 2018). In a disease map, interconnected pathway diagrams depict events from the level of molecular processes to higher levels like communication between cells and organs (Mazein et al., 2018; Ostaszewski et al., 2018; Mazein et al., 2021a). Approaches to the construction of disease maps advanced during past years, supported by the activity of the Disease Maps community (https://disease-maps.io) and formalised into best practices (Kondratova et al., 2018). However, there is still a lack of clear, formal and well-structured guidelines for main steps of developing a disease map in collaborative projects.

The need for such guidelines became evident during the development of the COVID-19 Disease Map (Ostaszewski et al., 2021), a large-scale project supported by an extensive community with more than 200 volunteer participants. The efforts of COVID-19 Disease Map Community would be greatly advanced with step-by-step guidelines to accelerate learning about the approach and creating high-quality resources.

There are a number of publications that can be used as a reference when constructing disease maps (Kondratova et al., 2018; Mazein et al., 2018). Moreover, brief and informative guideline articles about biocuration (Tang et al., 2019) and pathway construction (Hanspers et al., 2021) offer valuable introductory material for constructing disease-oriented resources. In particular, an article on construction of a Reactome-based specific knowledge repository (Varusai et al., 2020) proposes instructions on reuse of pathway diagrams in a specific context. However, this subject still lacks a comprehensive summary, encompassing the entire lifecycle of a disease map resource.

In this article, we offer guidelines for building diagrammatic systems biology knowledge repositories of disease mechanisms that are explorable online and transformable to computational models. In particular, we discuss planning and design of a disease map including best practices for defining the scope of the resource and communication with domain experts. We discuss the process of biocuration, the required granularity of the map and the required standard languages. Finally, we outline aspects of visual exploration and possible downstream bioinformatic analyses, and the strategy for maintaining the resource following FAIR principles (Wilkinson et al., 2016). We review the literature and examples for a particular area of disease map construction, structuring them into steps of a reference workflow. The methods described in this review are built on the experience of the previously developed disease maps including recently published resources (Ravel et al., 2020; Mazein et al., 2021a; Pereira et al., 2021; Zerrouk et al., 2022). Definitions of domain-specific terms are available at https://disease-maps.io/glossary.

## 2 Disease map development workflow

**FIGURE 1:**
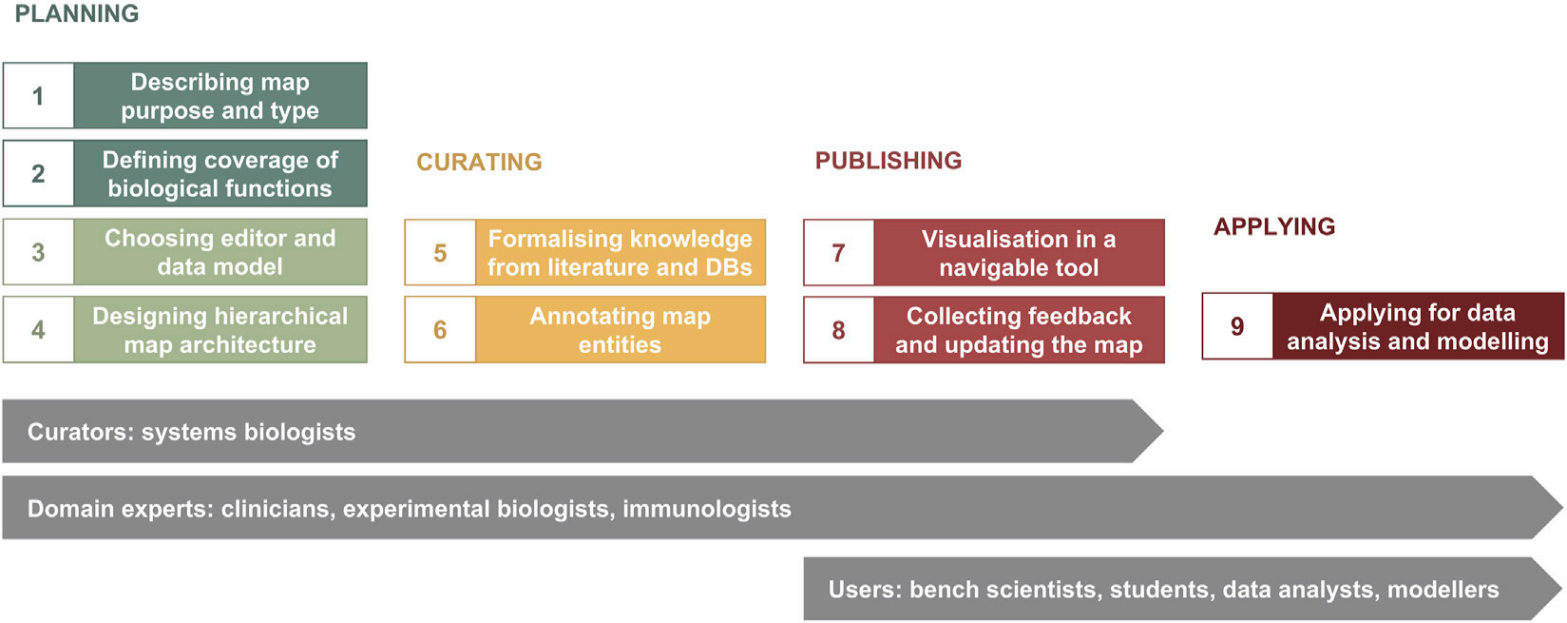
The map development workflow [based on Kondratova et al., 2018 (Kondratova et al., 2018)]. The workflow includes nine steps in four phases (please see in the text). Curators and domain experts are involved in planning and designing the map, then curators develop the map using a diagram editor of choice, advised by domain experts. After publishing online, data analysts, modellers and other users have access to the map for exploration and applications in translational projects, as well as domain experts suggest further evolving the map.

The major phases of map development are illustrated in Figure 1. The planning phase includes i) determining map purpose and type, ii) defining the coverage of biological functions, iii) choosing a graphical standard (data model) and an editor, and iv) designing map architecture (Figure 1, Steps 1–4). While these steps are shown in a sequence, they are often realised in parallel. Next, in the curation phase, available knowledge from literature and databases is encoded into a diagram with accompanying annotation of map entities (Figure 1, Steps 5 and 6). When the map is ready for publication, it is made available online in a navigable tool and updated based on feedback from domain experts—the publishing phase (Figure 1, Steps 7 and 8). The application phase depends on the goals of the project, expertise and resources available, and can include data visualisation, drug target exploration, computational modelling and hypothesis generation (Figure 1, Step 9).

### 2.1 Determining map purpose and type

At the initial step, it is important to define goals of the project and to determine the map type and its applications. Later, it will influence map’s format, design, size and coverage. The key questions at this stage are:

– What is the main focus? Is it one disease or multiple related disorders?
– What is the intended use of the map?
– What resources are available or needed?
– What is the timeline?
– Which domain experts (clinicians and biologists) can advise and revise the curation?

Examples of motivation, objectives and primary users for several disease map projects are shown in Table 1. Often they are aligned with the focus of a research centre so the developed mam supports related research. It also can be part of a large-scale translational project for integrating knowledge and future applications in interpreting data generated by the project.

**TABLE 1:** Examples of disease map purpose, intended use and expected primary users.

Primary purpose	Details	Examples	Primary users
Integrating knowledge	Visualisation, identifying gaps, educational purposes	Atlas of Cancer Signalling Networks Kuperstein et al. (2015a), Parkinson’s Disease Map Fujita et al. (2013), RA-Map Singh et al. (2020)	Research centres, students, modellers
Combining knowledge with external databases and tools	Exploring drug targets, text mining	COVID-19 Disease Map Ostaszewski et al. (2021)	COVID-19 research community, data analysts
Data analysis	Network-based analysis, omics and multi-omics data visualisation, interpretation and hypothesis generation	AsthmaMap Mazein et al. (2021a)	IMI U-BIOPRED (No 115010, https://www.imi.europa.eu) community, IMI eTRIKS (No 115446, https://www.etriks.org) community, data analysts
Computational modelling	Using maps as templates for building computational models, refining hypotheses and making predictions	Atherosclerosis Map Parton et al. (2019)	Research centres, modellers
Computational modelling combined with data analysis	Using maps as templates for building Boolean and hybrid modelling and data analysis	RA-Atlas Miagoux et al. (2021), Aghakhani et al. (2022); Zerrouk et al. (2022)	Research centres, modellers, data analysts

Intended map purpose might include one or several of the following objectives:

– integrating available knowledge:
  – reviewing and visualising disease mechanisms,
  – identifying gaps in knowledge,
  – educational purposes;
– combining knowledge with external databases and tools:
  – exploring drug targets,
  – text mining;
– analysis:
  – network-based analysis,
  – omics and multi-omics data visualisation, interpretation and hypothesis generation;
– computational modelling:
  – using maps as the basis/template for different types of models,
  – refining hypothesis,
  – making predictions.

Clarifying the motivation, objectives and intended applications is needed for the following steps about the coverage of biological functions. This can be documented in a brief project description document (https://disease-maps.io/template), or in a web page (e.g., https://disease-maps.io/projects), or in a project management platform such as FAIRDOMHub (https://fairdomhub.org).

### 2.2 Defining coverage of biological functions

Exchange with the disease domain experts allows to define the scope and boundaries of the map. Also, key reviews and figures that can help to create an initial skeleton of the map. The main tasks at this stage include:

– Identify priority pathways;
– Identify cell types, tissues and organs relevant to the pathology mechanisms;
– List the molecules known to be involved and drugs used in a given condition
– Define disease outcomes on the molecular, cellular and tissue levels, including subdivision into disease stages and subtypes, if applicable.

Such lists can be stored in a document shared and discussed with the domain experts, and maintained throughout the map development, e.g., https://disease-maps.io/template.

Often this information is available in relevant databases, like disease-focused pathways of Reactome (Reactome:R-HSA-1643685) including cancers (Reactome:R-HSA-5663202) and infectious diseases (Reactome:R-HSA-5663205, Reactome:R-HSA-9694516). Disease pathways of KEGG (https://www.genome.jp/kegg/disease), include Parkinson’s disease (KEGG:hsa05012) and COVID-19 (KEGG:hsa05171). The Open Targets Platform (https://platform.opentargets.org) [18] lists molecules relevant for various diseases, e.g., targets associated with allergic asthma (https://platform.opentargets.org/disease/MONDO_0004784/associations).

Outcomes of this step guide the curation efforts in steps 5 and 6 (Figure 1), and the coverage of the biological functions may change depending on available knowledge. In effect, this step is often revisited, so it is important to maintain versioning of the map planning document (https://disease-maps.io/template).

### 2.3 Choosing a graphical standard and an editor

**FIGURE 2:**
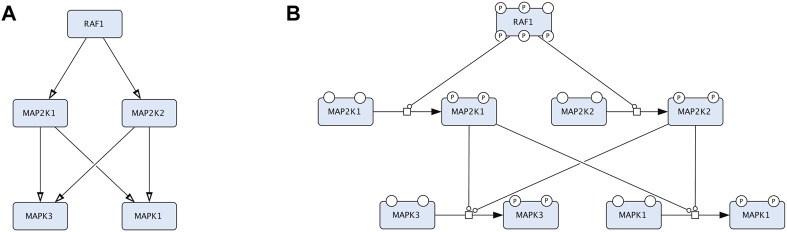
An example that compares two representations in CellDesigner that correspond to Activity Flow (Reduced Notation palette in CellDesigner) and Process Description (default palette in CellDesigner). The two diagrams represent the same biological events but in two conceptually different languages. **(A)**. The Process Description representation of the RAF-MEK-ERK signalling: the process of MEK1/2 phosphorylation is catalysed by RAF1 and the process of ERK1/2 phosphorylation is catalysed by the phosphorylated MEK1/2. **(B)**. The Activity Flow representation of the RAF-MEK-ERK signalling: the activity of RAF1 stimulates the activity of MEK1/2 (MAP2K1 and MAP2K2 in official HGNC names), and the activity of MEK1/2 stimulates the activity of ERK1/2 (MAPK3 and MAPK1 in official HGNC names).

Majority of disease maps are created in Systems Biology Graphical Notation (SBGN) (Le Novère et al., 2009) using the CellDesigner editor (https://www.celldesigner.org), using the SBGN Process Description (PD) and Activity Flow (AF) languages (Le Novère et al., 2009) (Figure 2). The Entity Relationship (ER) language (Le Novère, 2015) is not currently employed in developing disease maps.

Process Description (PD) (Rougny et al., 2019) and Activity Flow (AF) (Mi et al., 2015) are the two most used languages of the Systems Biology Graphical Notation (SBGN) standard (www.sbgn.org) (Le Novère et al., 2009) (Figure 2). More information about the SBGN standard are available in the “SBGN Learning” materials at https://sbgn.github.io/learning and the “Figure to SBGN” page at https://sbgn.github.io/figuretosbgn.

CellDesigner (https://www.celldesigner.org) is a diagram editor that is frequently used for developing disease maps. It supports extensive diagrams and follows SBGN logic and makes it possible to draw SBGN-compatible diagrams in Process Description and Activity Flow type of languages using the default CellDesigner’s palette or its Reduced Notation palette. Alternative solutions include the open-source web-based Newt Editor (Balci et al., 2020), the open-source VANTED editor with its SBGN-ED add-on (Czauderna et al., 2010) and the freely available yEd Graph Editor from yWorks (https://www.yworks.com/products/yed). Each tool has its own advantages. Newt offers support for the latest 0.3 SBGN-ML (Bergmann et al., 2020), SBGN bricks (Rougny et al., 2021), advanced automatic layout (Balci and Dogrusoz, 2022), semantic validation of maps, conversion to and from CellDesigner (Balaur et al., 2020) and SBML formats, and an easy switch to alternative colour schemes. SBGN-ED allows map validation (Czauderna et al., 2010), conversion from KEGG and SBML formats and PD-to-AF conversion (Vogt et al., 2013). yEd has an SBGN palette and SBGN layout tool (Siebenhaller et al., 2018), offers intuitive drawing and can support very extensive graphs. The AsthmaMap and the Cystic Fibrosis Map (Pereira et al., 2021) were developed in the yEd Graph Editor, then converted to the SBGN-ML format via the ySBGN tool (Balaur et al., 2022) and then to CellDesigner and MINERVA.

The choice of an editor is also connected to the visualisation platform used (Figure 1, step 7). The MINERVA and NaviCell (Kuperstein et al., 2013; Bonnet et al., 2015) platforms by design are compatible to CellDesigner’s XML. The MINERVA Platform also accepts SBGN-ML files and SBML files with layout (Hoksza et al., 2019a), and can therefore be used with Newt, SBGN-ED, as well as with yEd in combination with the ySBGN converter. Importantly, the platform also supports export to these formats, allowing to review, improve and reupload disease map diagrams.

### 2.4 Designing map architecture

Depending on the goals of the project, map coverage and the resources available, there are several possibilities for designing map architecture and for step-by-step progress including starting from PD or AF layer and working with a single diagram or a set of sub-pathways (Table 2).

**TABLE 2:** Examples of map architecture from a single diagram to a hierarchically-organised multi-layered structure.

#	Disease map	Architecture	Format	Top-level view
a	AlzPathway Mizuno et al. (2012), Ogishima et al. (2016), RA-Map Singh et al. (2020)	Single diagram	Process Description	None
b	FluMap Matsuoka et al. (2013)	Single diagram	Process Description	None
c	Cystic Fibrosis Map Pereira et al. (2021)	Single diagram	Activity Flow	None
d	Parkinson’s Disease Map Fujita et al. (2013); ACSN Kuperstein et al. (2015a)	Hierarchical	Process Description, Activity Flow	Illustrative
e	ACSN Kuperstein et al. (2015a); RA-Atlas Zerrouk et al. (2022), COVID-19 Disease Map Ostaszewski et al. (2021)	Hierarchical	Process Description	Integrating diagram^a^
f	AsthmaMap Mazein et al. (2021a)	Hierarchical	Process Description, Activity Flow	Integrating diagram^a^

^a^ The integrating diagram has elements that represent lower-layer sub-pathways and this way unites them into a single virtual map.

**FIGURE 3:**
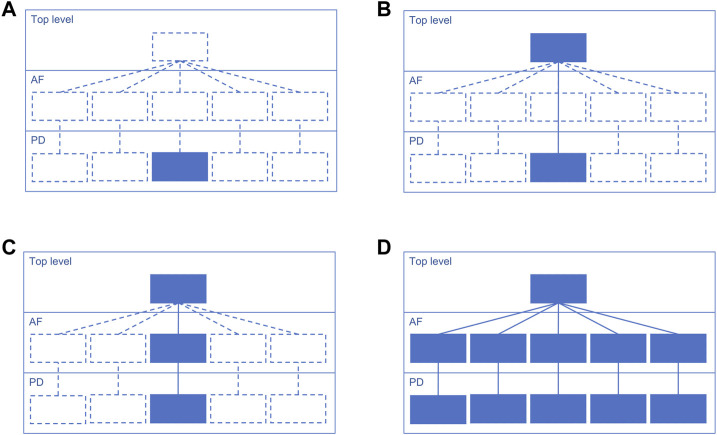
A recommended way to gradually build a hierarchical structure starting from a single PD diagram and step by step making the map architecture more complex. Each map version works as a fully functional complete resource. **(A)**. Starting with a priority sub-pathway in PD. **(B)**. Building a top-level view with key mechanisms partly or entirely represented in the PD diagram. **(C)**. Automatically or manually creating an AF diagram that matches the content of the PD diagram. **(D)**. Extending the content to multiple diagrams with one integrating top-level view diagram that represents more detailed AF and PD layers.

Suggested starting design is a PD diagram of mechanisms with accompanying top-level view of disease mechanisms. This design focuses on the prioritised pathways, allows reuse of the PD layer from other projects, and helps communicating with domain experts via the top-level view (Figure 3). Please note that each map version (A, B, C and D) is a functional, complete resource. Potentially the AF layer can be created automatically from the PD layer using CaSQ (Aghamiri et al., 2020) or a PD-to-AF converter (Vogt et al., 2013). More options for building a multi-layered structure and example architectures of the published maps are available at https://disease-maps.io/architecture.

#### 2.4.1 Modularisation

Modular design of a disease map means developing diagrams that are manageable and reusable (Mazein et al., 2021b) for faster curation, reuse in other projects and downstream use in computational modelling (Cooling et al., 2016; Niarakis et al., 2021; Niarakis et al., 2022). A module is a diagram part that is self-contained and can be developed and maintained independently while being included in a larger network. Modularisation can be applied at different scales including signalling subnetworks, e.g., the MAPK cascade, and on a larger scale, e.g., cell-specific pathways in mast cells, eosinophils and macrophages in asthma (AsthmaMap, https://asthma-map.org/mr).

#### 2.4.2 Examples

AlzPathway (http://alzpathway.org) (Mizuno et al., 2012; Ogishima et al., 2016) catalogues signalling pathways in Alzheimer’s disease as the first comprehensive map of a particular disease. It is a single PD diagram in CellDesigner with an overview image highlighting the included pathways.

The Parkinson’s Disease Map (Fujita et al., 2013) was originally designed as a single diagram integrating disease-relevant pathways, e.g., mitochondrial dysfunction, calcium homeostasis, synaptic pathobiology, α-synuclein misfolding, failure of protein degradation systems, apoptosis and neuroinflammation. Later a top-level view was added in MINERVA for easier overview and navigation (“SHOW OVERVIEW” button), and a number of pathways were added as underlying PD diagrams (https://pdmap.uni.lu/minerva).

The FluMap (Matsuoka et al., 2013) is a comprehensive map of the influenza A virus replication cycle. It is available as a single PD diagram with a simplified version in an AF-compatible format developed in the Reduced Notation in CellDesigner.

The Atlas of Cancer Signalling Networks (ACSN) (Kuperstein et al., 2015a) is a collection of large-scale PD diagrams that cover mechanisms of cancer and include cell cycle and DNA repair, cell survival, regulated cell death, telomere maintenance, epithelial-mesenchymal transition (EMT) and senescence, invasion and motility, and many more (https://acsn.curie.fr/ACSN2/maps.html). These modules are also available as part of a single extensive map (https://acsn.curie.fr).

The AsthmaMap includes three layers: the detailed Biochemical Mechanisms layer in PD (https://asthma-map.org/bm), the intermediate Molecular Relations layer in AF (https://asthma-map.org/mr) and the top-layer Cellular Interactions view in AF-compatible format (https://asthma-map.org/ci) (Mazein et al., 2021a). The three layers are interconnected and presented in a hierarchically-organised map in MINERVA (https://asthma.uni.lu).

The Rheumatoid Arthritis Map (RA-Map) was initially designed as a single Process Description map (Singh et al., 2020) and then evolved into the Rheumatoid Arthritis Atlas (RA-Atlas), a collection of interconnected diagrams with an access point via a top-level view (Zerrouk et al., 2022). The RA-Atlas includes an updated version of the global RA-Map (Singh et al., 2020) covering relevant metabolic pathways and cell-specific molecular interaction maps for CD4^+^ Th1 cells, fibroblasts, and M1 and M2 macrophages.

The COVID-19 Disease Map is a collection of interconnected Process Description diagrams developed by a community-driven effort in the answer to the SARS-CoV-2 pandemic (https://covid.pages.uni.lu). The map gathers diagrams developed by curators using CellDesigner and VANTED software, describing mechanisms of virus cycle in the host cells, its interactions with host molecular pathways, and the mechanisms of host response to the infection. These diagrams are organised into one hierarchical map via a top-level view diagram (https://covid19map.elixir-luxembourg.org/minerva). Notably, the COVID-19 Disease Map Community involves diagram curators of the Reactome and WikiPathways platforms, hosting their SARS-CoV-2-related diagrams.

#### 2.4.3 Choice and implementation of a map architecture

The choice of a map design should match the biological coverage of the map (Figure 1, step 2) and the overall progress of the project. The design should be adapted in case of a change in the coverage, timeline or available resources, as the stages introduced in Figure 3 are flexible. Considering the discussed examples, a multi-layered and modularised hierarchically-organised map should be considered as a default design (Figure 3D). Depending on a project, the focus can be only on one layer, PD or AF, and the top-level view is optional (Figures 3A, B). Though, with adequate resources available, it is recommended to work on a multi-layered hierarchical map as the state-of-the-art practice. The main advantages of this hierarchically-organised structure (Table 3):

– rich content and detailed representation supports different types of visualisation and different types of computational modelling;
– a possibility of reusing available resources;
– compatibility with similar disease map projects, map parts are easy to reuse in new projects;
– a top-level view of disease hallmarks helps communicating with domain experts.

**TABLE 3:** Comparison of the top-level view vs. Activity Flow vs Process Description layers in a hierarchically-organised map. Resources: KEGG: Kyoto Encyclopaedia of Genes and Genomes (KEGG) (Kanehisa and Goto, 2000), OmniPath (Türei et al., 2016), SIGnaling Network Open Resource (SIGNOR) (Licata et al., 2020), Reactome (Gillespie et al., 2022), Recon and ReconMap (Thiele et al., 2013; Noronha et al., 2017), PANTHER (Mi et al., 2019).

Features	Top layer: top-level view	Intermediate layer: AF	Detailed layer: PD
SBGN language	Activity Flow or free format^b^	Activity Flow	Process Description
Connectivity	Interactions	Interactions	Processes
Granularity	Least detailed: focus on key molecules and cell types	Intermediate level of details	Most detailed, the level of processes
Compatible databases	Not available^a^	KEGG signalling pathways, BioCarta, OmniPath, SIGNOR	Reactome, PANTHER, Recon X, KEGG metabolic pathways
Application/Use	Communication with domain experts for defining disease hallmarks	omics visualisation, network analysis, Boolean modelling, integration with machine learning inferred networks	omics visualisation, network analysis, ODE modelling, Boolean modelling (via CaSQ)

^a^ The closest similar visualisations are figures with an overview of disease mechanisms in review papers, representations with cell types, tissues and organs shown.

^b^ By free-format here we mean flexible representation with one necessary function: all lower-layer sub-pathways in a hierarchically-organised structure are represented by an element on the top-level view and this way integrate them into a single virtual map.

Finally, other design alternatives are possible, e.g., with more high-level layers or using other standard languages. For instance, the disease map approach supports the concept of adverse outcome pathways (AOPs) in the field of immunotoxicology (paper in preparation).

### 2.5 Formalising knowledge from literature and databases

In the knowledge formalisation step, relevant causal interactions and processes are taken from selected sources including disease-relevant scientific papers and existing representations in pathway databases. In case of scientific papers, causal interactions and processes are manually identified, integrated and encoded into a diagrammatic representation (Kondratova et al., 2018). In case of pathway databases, relevant causal interactions are manually reviewed and contextualised for a specific disease. Knowledge about molecular processes relevant in the context of a particular disease need to be represented in a standard way, for example, receptor activation, phosphorylation, complex formation, positive and negative regulation of transcription, transport, glycosylation, and more. Knowledge is transformed from textual descriptions and figures in scientific articles into a standardised diagrammatic representation. This representation is also machine-readable and can be further converted into executable computational models.

Knowledge about disease mechanisms is formalised in the SBGN standard within two conceptually different languages, AF and PD. AF is focusing on molecular activities and their sequential connectivity, and PD is focusing on the detailed description of the involved molecular processes (see Table 3; Figure 2). AF is compact and is suitable for overview diagrams and for highlighting key mechanisms. PD is detailed and is better for comprehensive representation of molecular events.

**FIGURE 4:**
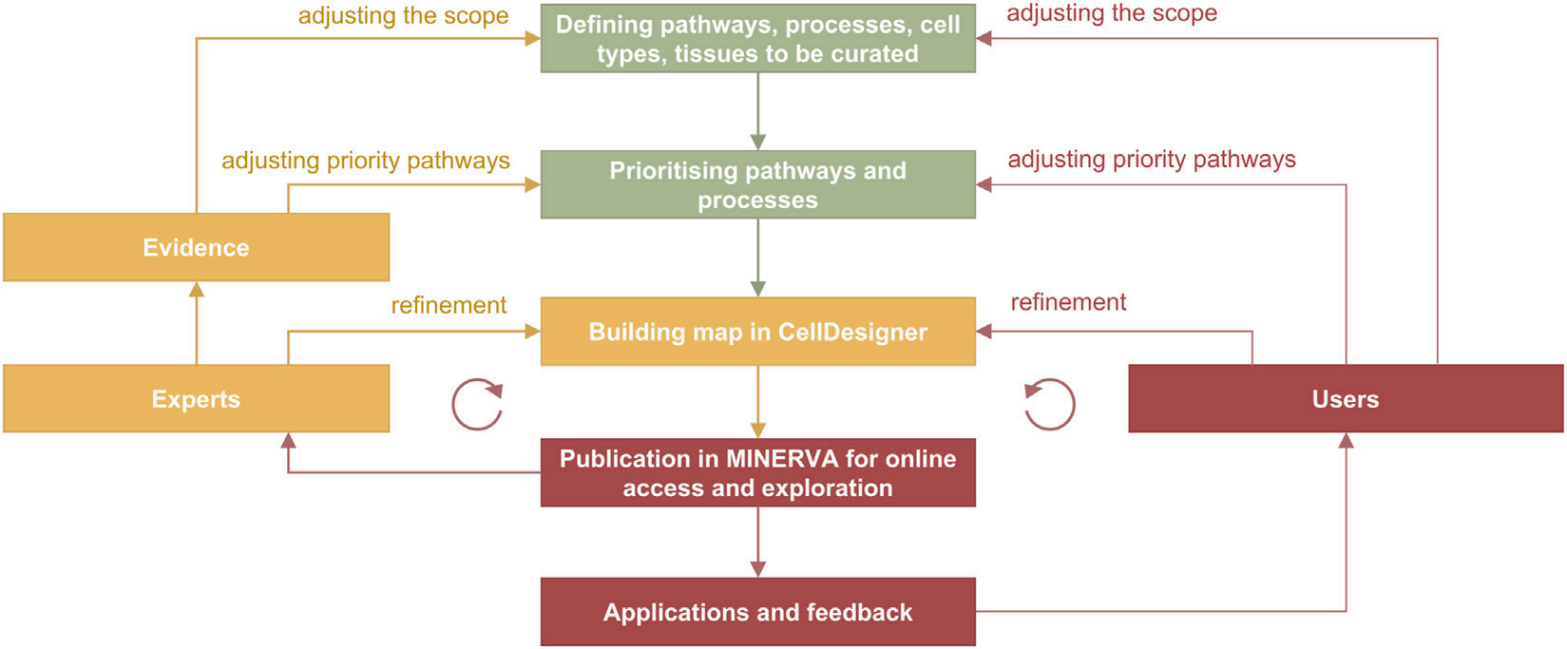
Curation and refinement cycle in disease map development. In green are the stages from the planning phase, in yellow from the curation phase and in red from the publication and application stages. The cycle on the left includes feedback from domain experts involved in the development, and the cycle on the right includes feedback from users. Evidence are integrated from PubMed and PubMed Central searches, from working with pathway databases such as Reactome (Gillespie et al., 2022), Recon (Thiele et al., 2013; Noronha et al., 2017) and PANTHER (Mi et al., 2019), and annotation databases such as UniProt (https://www.uniprot.org) and ChEBI (https://www.ebi.ac.uk/chebi).

In the curation cycle (Figure 4) information is manually integrated from different sources such as review papers and discussions with domain experts, producing lists of relevant molecules, pathways and cell types. Prioritised pathways are curated in CellDesigner from literature or pathway databases, and visualised in MINERVA for use and review, leading to updates in CellDesigner.

The knowledge formalisation and integration works in a similar way to how we build a review paper but, in this case, it is structured and systematic to follow the rules of the graphical standard and to ensure the connectivity in the network. Disease map curation can be seen as a graphical review of relevant disease mechanisms and can be compared to a combination of scoping, rapid and systematic reviews (Grant and Booth, 2009). A scoping review, corresponding to step 2 of the map development process (Figure 1), determines the coverage of biological functions, defines disease hallmarks, and identifies knowledge gaps. Rapid and systematic reviews, corresponding to steps 5 and 6 (Figure 1), narrow down the focus and collect evidence from literature and databases for drawing and annotating diagrams in an editor. These steps are usually performed by a dedicated curator with advice and feedback from a panel of domain experts.

This “graphical review” requires following diagram-specific knowledge representation formats. It demands consistency in using a standardised graphical language and describing connectivity between diagram elements that may span beyond the currently curated article. For example, if a paper states that IL6 activates epithelial cells, it is important to identify the relevant protein (IL6, UniProt:P05231), identify receptors involved (IL6RA, IL6ST, IL6RB), and describe the following signalling pathway, which may require looking for additional articles.

More details on knowledge formalisation for disease maps are available at https://disease-maps.io/formalisation: the webpage provides examples of text-to-SBGN and figure-to-SBGN transformation, examples of search queries in PubMed and PubMed Central, and describes text-mining capabilities of Europe PMC (Ferguson et al., 2021).

A map project can include multiple collaboration and exploration pipelines. An extended example of map development and application ecosystem is available for the COVID-19 Disease Map community in Figure 1 in the 2021 publication (Ostaszewski et al., 2021).

### 2.6 Annotating map entities and interactions

Two objectives during the annotation step are identifying map components and providing evidence for interactions. Identification assigns stable identifiers or ontology terms to map components to uniquely define them. This ensures interoperability with systems biology tools and external databases. Providing evidence for interactions defines the context of a given interaction, e.g., a specific cell type, tissue, organism, or relevant pathology. Contrary to components, these annotations are not unique but interaction-specific, and require evidence from a relevant source, e.g., scientific publications, curated pathways, or interaction databases.

#### 2.6.1 Annotating entities

Different types of map entities are shown in Table 4 together with recommended annotation.

**TABLE 4:** Recommended annotation of map entities. Resources: HUGO Gene Nomenclature Committee (HGNC, https://www.genenames.org), UniProt (https://www.uniprot.org), Complex Portal (https://www.ebi.ac.uk/complexportal) (Meldal et al., 2022), ChEBI (https://www.ebi.ac.uk/chebi), PubChem (https://pubchem.ncbi.nlm.nih.gov), ChEMBL (https://www.ebi.ac.uk/chembl), DrugBank (https://go.drugbank.com), Cell Ontology (https://www.ebi.ac.uk/ols/ontologies/cl), BRENDA (https://www.brenda-enzymes.org), Cellosaurus (https://www.cellosaurus.org), GO (http://geneontology.org), MeSH (https://www.ncbi.nlm.nih.gov/mesh).

Biochemical entity	Naming^a^	Identifier
Protein	HGNC official symbol^b^	UniProt/HGNC
RNA	HGNC official symbol	HGNC
Gene	HGNC official symbol	HGNC
Metabolite	ChEBI/PubChem recommended name	ChEBI/PubChem
Drug	ChEBI/PubChem recommended name^c^	ChEBI/PubChem/ChEMBL/DrugBank
Complex	Specific name from literature or listing complex components: Element A:Element B	Not required. If available: Complex Portal
Compartment	Appropriate term from Cell Ontology, BRENDA, Cellosaurus, or a specific name from literature	Cell Ontology/BRENDA/Cellosaurus
Phenotype (biological processes)	Appropriate Gene Ontology (GO) Biological Process (BP) term if available^d^	GO Biological Process
Phenotype (symptom, disease state)	Appropriate MeSH term if available^d^	MeSH

^a^ Flexible if an identifier is provided.

^b^ This way the protein will be annotated on upload to MINERVA but UniProt will be assigned only if there are no multiple proteins that correspond to that name.

^c^ Non-standard names are allowed as long as the annotation is provided: for example, “ERK1” instead of “MAPK3” (UniProt:P27361) or “PGE2” instead of “prostaglandin E2” (ChEBI:15551).

^d^ As in the literature or as suggested by the domain experts.

Proteins, RNAs and genes should be named according to the official symbols of the HUGO Gene Nomenclature Committee (HGNC, https://www.genenames.org). For example, while COX1 is a commonly used name, PTGS1 symbol is recommended. This provides consistency in naming and allows skipping manual addition of other stable identifiers in CellDesigner, because HGNC symbols are recognised by the MINERVA annotation tool on upload.

Annotation of complexes is optional because they can be identified by their components. However, it is recommended to name them by listing the molecules involved, for example, “FCER1A: FCER1G:MS4A2”, or using specific names if available. When annotating, complex-specific annotations are recommended, e.g., GO Cellular Component (CC) or Complex Portal identifiers (Meldal et al., 2022) (https://www.ebi.ac.uk/complexportal). For instance, GO CC term “Fc-epsilon receptor I complex” (GO:0032998) for the complex above.

Phenotypes or other elements representing higher order processes should be annotated with Gene Ontology (GO) Biological Process (BP) terms () (Gene Ontology Consortium et al., 2021) or MeSH terms (https://www.ncbi.nlm.nih.gov/mesh). GO BP is recommended for annotating components describing biological processes and MeSH terms are recommended for diseases-related terms.

Metabolites (“simple chemical” in SBGN, or “simple molecule” in CellDesigner) are identified via the Chemical Entities of Biological Interest database (ChEBI, https://www.ebi.ac.uk/chebi) (Hastings et al., 2016). Alternatively, PubChem (https://pubchem.ncbi.nlm.nih.gov) (Kim et al., 2021), Human Metabolome Database (https://hmdb.ca) (Wishart et al., 2007) or LIPID MAPS (https://www.lipidmaps.org) (Sud et al., 2007) can be used. Automatic MINERVA annotation works for standard ChEBI names, for example, “arachidonic acid” (CHEBI:15843), however in other cases annotations need to be provided manually.

An extended description of annotation for disease maps at offers suggested annotation of diagram information and specific details of annotation fields in CellDesigner.

Other useful sources on the topic of annotation are the recommendations of the logical modelling community (Niarakis et al., 2021), the Reactome data model and pathway development practices (Varusai et al., 2020), the rules suggested for creating reusable pathway models (Hanspers et al., 2021), the documentation of the MI2CAST format (Minimum Information about a Molecular Interaction CAusal STatement) (Touré et al., 2021b) and the protocol for high-quality metabolic reconstruction (Thiele and Palsson, 2010).

#### 2.6.2 Annotating interactions

Scientific articles contain evidence that confirm the existence of molecular interactions in the context of a specific disease. Each interaction should be annotated with stable identifiers of relevant articles, a PubMed ID or a DOI. If pathway interactions are used, their identifiers can be used instead. Although some guidelines suggest a minimal number of references confirming an interaction (Kondratova et al., 2018), this should be adapted to the literature coverage of a given disorder.

Importantly, often the relevance of molecular processes in the context of a disease can only be confirmed with a combination of references. For example, one paper can confirm that a specific process is involved in a disease, and another paper will describe details of this process. Only together they offer strong evidence for the interaction.

Some molecular processes related to the disease of interest can be detected in non-human organisms, typically in well-established rodent models for that disease. For parts built based on evidence from cell or animal models, it is important to annotate the corresponding interactions. As proteins, RNA and genes are named using HGNC symbols (human gene symbols), it is recommended to annotate interactions with the NCBI taxon ID of the non-human organism in which the molecular process was determined. For example, if the phosphorylation of STAT3 by JAK2 was determined in mice, the NCBI Taxonomy ID “10090” (NCBI:txid10090) annotation should be added to the interaction.

As most of disease-specific evidence comes from the literature, annotating map interactions requires maintaining a large repository of articles. Such a repository should be stored in a reference manager like Zotero (https://www.zotero.org) or Mendeley (https://www.mendeley.com). Articles should be archived while curating and annotating interactions, preferably in a structure mirroring the organisation of the map. Overall, it is important to check that all interactions in the diagram are annotated with evidence. Automated validation is strongly advised, for instance by using the validators available in the MINERVA Platform.

### 2.7 Visualising and analysing the maps

Systems biology diagrams of disease maps are developed for online interactive exploration and visual analytics to bridge the gap between the domain experts and bioinformatics experts. The maps can also be used as a part of larger bioinformatic workflows. For example, they can be transformed into a graph database for scalable exploration and analysis, for instance via Neo4j (Section 4), or integrated into a shared network repository like the NDEx platform (Pratt et al., 2015). Their content can be transformed with the use of the CaSQ tool for Boolean modelling (Section 2.4; Table 3), or for analysis of network topology with the help of tools such as Cytoscape (Shannon et al., 2003). However, the reminder of the section focuses on the visual exploration aspect of disease maps, using a dedicated online platform.

The MINERVA Platform (Gawron et al., 2016; Hoksza et al., 2019a; Hoksza et al., 2019b) enables easy online access to and visual interactive exploration of disease maps. Examples are:

– Parkinson’s Disease Map (https://pdmap.uni.lu);
– AsthmaMap (https://asthma.uni.lu);
– Rheumatoid Arthritis Map (https://ramap.elixir-luxembourg.org);
– Cystic Fibrosis Map (https://cysticfibrosismap.github.io);
– COVID-19 Disease Map (https://covid19map.elixir-luxembourg.org).

Video tutorials are available for introducing key MINERVA functionalities with the AsthmaMap as an example: navigating the maps, adding comments, exploring drug targets and visualising data (https://asthma-map.org/tutorials).

Maps can be uploaded to MINERVA in several formats including CellDesigner SBML, SBML + layout + render, SBGN-ML and GPML (Hoksza et al., 2019a). The conversion module within the MINERVA Platform allows export to these formats as well. On upload users can choose from CellDesigner’s view and SBGN view.

Automatic annotation can be selected on upload. As discussed above, with HGNC names used for proteins, mRNAs and genes, the MINERVA Platform automatically adds links to relevant external databases.

Map browsing and zooming works in MINERVA in the Google Maps manner. For multi-layered maps, interconnected diagrams are organised into a single entity via the submaps function and are visible from the top-level diagram in the SUBMAPS tab (https://asthma-map.org/tutorials/#navigating-the-asthmamap). Search functionality queries all connected maps.

Data can be uploaded as a tab-separated file (https://minerva.uni.lu/doc/examples). This way, omics data (for example, differential expressed genes) can be converted into a gradient of colours on the map (https://asthma-map.org/tutorials/#visualising-data). Multiple datasets can be visualised simultaneously and compared, including a multi-omics combination (e.g., proteomics and metabolomics), or time series data with different time points visualised as separate datasets.

Drug target search connects via online queries to DrugBank (https://www.drugbank.ca) (Wishart et al., 2018) and ChEMBL (https://www.ebi.ac.uk/chembl) (Gaulton et al., 2012; Bento et al., 2014) databases and allows highlighting all entities affected by a drug (https://asthma-map.org/tutorials/#exploring-drug-targets).

Moreover, MINERVA API and plugins (Hoksza et al., 2019b) allow the implementation of customised visual exploration and analytics pipelines.

An alternative web-based tool is NaviCell (Kuperstein et al., 2013; Bonnet et al., 2015) developed primarily to support the ACSN project. It offers functionalities for visualising omics data for performing functional analysis of the maps, and for computing aggregated values for sample groups and their visualisation on the maps (https://navicell.vincent-noel.fr). A video tutorial on ACSN in NaviCell is available at https://acsn.curie.fr/documentation.html#video.

### 2.8 Collecting feedback and updating the map

When the first complete version of a map is available online, it is advisable to request feedback from domain experts regarding the coverage of disease hallmarks, the depth and quality of pathways included, accuracy and adequacy of the disease mechanisms representation. This input can be collected in the form of 1) free-format notes, 2) suggested publications, or 3) directs comments on the map in MINERVA (https://asthma-map.org/tutorials/#commenting).

It is also suggested to have an automatic check of any inconsistencies in curation and annotation. This can be done using Neo4j-based tools (see the “Disease map management in the Neo4j graph database” section) or the automated verification in the MINERVA Platform (https://minerva.pages.uni.lu/doc/admin_manual/v16.0/index/#configure-automatic-verification).

### 2.9 Applying the resource

Map applications can play a role in the development process, as analysis and computational modelling may identify missing feedback loops or analytical endpoints, key pathways, or larger gaps in the network. Therefore, considering the use-cases of a map can help with its curation, revision and refinement (Ostaszewski et al., 2021).

One of map applications is omics data visualisation for its interpretation, hypothesis generation, and planning validation experiments. Another is exploration of drug targets in MINERVA using the corresponding functionality (https://asthma-map.org/tutorials). Finally, computational modelling allows to transform a static diagrammatic representation into a dynamic view of disease progression to be used for refining hypotheses and for predictions.

Demonstrated applications of disease maps include:

Data visualisation:
  – omics data visualisation (Kuperstein et al., 2015b; Satagopam et al., 2016; Mazein et al., 2021a);
  – analysis of cell-specific mechanisms using single-cell expression data (Ostaszewski et al., 2021);
  – RNA-Seq-based analysis of the activity of transcription factors (Ostaszewski et al., 2021);
Network-based analysis:
  – network analysis and representing map-based molecular signatures for sub-groups of patients in cancer (Kuperstein et al., 2015b; Jdey et al., 2016);
  – structural network analysis together with omics data to rationalise the synergistic effects of drugs towards the design of complex disease stage-specific druggable interventions in preclinical studies (Monraz Gomez et al., 2019);
  – network-based analysis and prediction of epithelial-to-mesenchymal-like transition mechanisms followed by experimental validation with the use of an animal model, a transgenic mice (Chanrion et al., 2014);
Computational modelling:
  – creating computational models based on disease maps, for example, for rheumatoid arthritis (Miagoux et al., 2021; Aghakhani et al., 2022) and atherosclerosis (Parton et al., 2019);
  – creating causal-interaction networks based on a disease map (Touré et al., 2021a);
In preclinical studies with follow-up validation experiments:
  – in preclinical studies in cancer (Chanrion et al., 2014; Jdey et al., 2016; Monraz Gomez et al., 2019) with validation of the proposed hypothesis by follow-up experiments;
Fusing disease maps with other solutions:
  – integrating disease maps with machine learning inferred networks (Miagoux et al., 2021);
  – identifying new crosstalks between pathways from combined analysis of interactions and text mining datasets (Ostaszewski et al., 2021).

## 3 Long-term sustainability of disease maps

Developing a disease map requires a significant amount of work thus it is important to ensure its long-term sustainability and use. First, the map should be deployed on a stable IT infrastructure for uninterrupted access to the content. This can happen via institutional resources or via a third party, such as the open-access map hosting service offered by ELIXIR Luxembourg (https://elixir-luxembourg.org/services/catalog/minerva). Second, a universal programmatic interface should be set up allowing reproducible queries to the disease map contents. The performance of the already available MINERVA API can be further improved by deployment of graph databases, supporting large and complex queries. Finally, a map should be set up as a FAIR resource (https://www.go-fair.org/fair-principles) (Wilkinson et al., 2016), ensuring its long-term reuse. Following sections discuss the topics of graph database deployment and map FAIRification.

## 4 Disease map management in the Neo4j graph database

**FIGURE 5:**
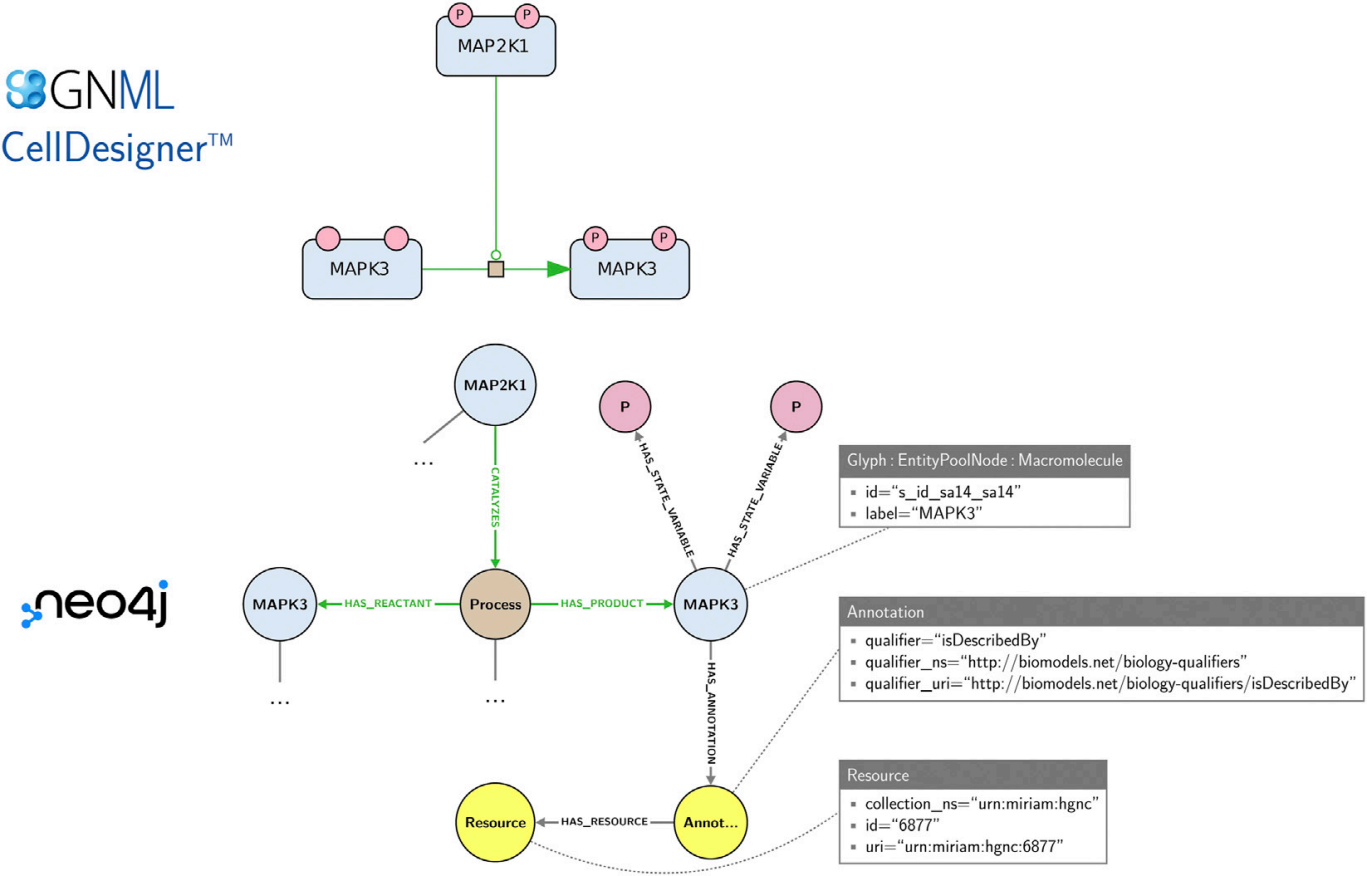
An SBGN map and an excerpt of its corresponding Neo4j graph built using the StonPy software. SBGN nodes (e.g., proteins, states variables) are modelled using Neo4j nodes, while SBGN arcs (e.g., catalyses, production arcs) and relationships between concepts are modelled using Neo4j relationships (edges). Neo4j nodes are labelled (e.g., “Glyph”, “Macromolecule”) and may contain key-value pairs (e.g., the pair “label”/“MAPK3”). Additionally, annotations are stored in a structured form that can be easily queried. In the complete model (not shown), SBGN arcs are additionally modelled using Neo4j nodes, as they may contain SBGN nodes themselves.

Maps offer visual representations primarily intended for human comprehension. For their exchange and edition, they are stored in machine-readable formats, e.g., CellDesigner SBML and SBGN-ML. While these formats are well suited for storing and exchanging graphical layout information, they do not offer a convenient solution for the network-based management (integration, querying, exploration) and analysis of collections of maps. The Neo4j graph database was proposed for an efficient management of large-scale highly-interconnected heterogeneous biological data (Lysenko et al., 2016), including biological pathway representations in systems biomedicine like Reactome (Fabregat et al., 2018), IntAct (Del Toro et al., 2022) and Recon2Neo4j (Thiele et al., 2013; Balaur et al., 2016). Collections of SBGN maps may also be stored and explored in a Neo4j database using the StonPy software (Rougny et al., 2023), which builds a Neo4j graph from an SBGN-ML map or a CellDesigner map converted to SBGN-ML using cd2sbgnml (Balaur et al., 2020). Data model of StonPy is close to the visual representation of maps and that is easy to learn. In this model, SBGN nodes (e.g., proteins, states variables) are modelled using Neo4j nodes, while SBGN arcs (e.g., catalyses, production arcs) and relationships between concepts are modelled using Neo4j relationships (edges). Annotations are also stored and can be easily queried. Figure 5 shows an example of a map and an excerpt of its corresponding Neo4j graph as built by StonPy.

The StonPy workflow was used to perform a comparative analysis of integrated biological pathways in several major resources including the Atlas of Cancer Signalling Networks (ACSN), PANTHER, and Reactome (Mazein et al., 2021b; Rougny et al., 2021). The cd2sbgnml (Balaur et al., 2020) and StonPy tools were also used to develop the dedicated C19DM-Neo4j graph database component (https://c19dm-neo4j.covid.pages.uni.lu), which integrates the biological content of the COVID-19 Disease Map diagrams available in MINERVA.

## 5 FAIR principles for disease maps available in MINERVA

Disease Maps approaches evolve together with other systems biology communities such as COMBINE (Hucka et al., 2015), CoLoMoTo (Niarakis et al., 2021), SysMod (Dräger et al., 2021), SBGN (Le Novère et al., 2009), SBML (Keating et al., 2020) and FAIR (Wilkinson et al., 2016) to enhance the accessibility and reusability of the resources (Niarakis et al., 2022).

The FAIR (Findable, Accessible, Interoperable and Reusable) principles (https://www.go-fair.org/fair-principles) establish criteria to assess and improve the reuse of scientific resources including data, metadata and infrastructure.

The underlying MINERVA Platform provides a set of functionalities that addresses several elements of FAIR:

– unified API calls to access the standardised content of all diagrams on the platform;
– functionality for translating between systems biology standards;
– authentication and authorisation procedures for user management;
– explicitly mentioning the reuse licence (for example, the COVID-19 Disease Map diagrams are released in MINERVA under the CC-BY 4.0 licence).

More specifically, MINERVA conforms to FAIR expectations in the following ways:

Findability: The content of the disease maps diagrams in MINERVA is annotated with a range of information elements from initial publications, connected to well-established biomedical resources (e.g., HGNC, UniProt, ChEBI, ChEML, PubMed, etc.) using standard identifiers. The maps are openly available online (e.g., at https://covid19map.elixir-luxembourg.org) and indexed in all major search engines.

Accessibility: The content of biological maps in MINERVA is accessible easily online via standard web protocols. Moreover, MINERVA provides uniform API calls to access the content of the diagrams and can provide required authentication (credentials: user ID and password) and authorisation procedures where necessary.

Interoperability: The biological content in MINERVA follows XML-based systems biology standards such as CellDesigner SBML; translation to other systems biology standard formats is also possible via MINERVA. Moreover, the content of maps is rich in metadata through annotations with information from initial publications connected to biomedical resources as recommended in Table 3, and there are ongoing efforts to connect the content to the Bricks Ontology (Rougny et al., 2021).

Reusability: The biological content of disease map diagrams in MINERVA is released under a specific data usage licence, e.g., under the CC-BY 4.0 usage licence for COVID-19 Disease Map (Ostaszewski et al., 2021). The biological diagrams are primarily edited using CellDesigner, thus complying with the incorporated rules for molecular mechanism representation, and are annotated with various types of information including references to publications (PMIDs) and standard identifiers for molecular entities (UniProt IDs for proteins, ChEBI IDs for metabolites, etc.).

By conforming to most aspects of the FAIR principles, the utility of disease maps available in MINERVA to the systems biomedicine community is greatly enhanced. While there is always room for improvement, and particularly in the domains of versioning and obsoleting policies, the application of FAIR principles throughout the development process of disease maps ensures the long-term relevance of the resource.

## 6 Conclusion

Knowledge of disease mechanisms is dispersed across many publications and domains. Systematic reconstructions of disease mechanisms with the help from disease domain experts allow building reference resources where fragmented information is integrated into disease maps as conceptual disease models. These maps are applied for interpreting newly generated omics data and for computational modelling in order to study diseases as complex systems. For this reason, disease maps need to be constructed in a responsible way, based on a formal, structured and detailed protocol enriched with illustrative examples. This will ensure high-quality and consistency, as well as enable efficient coordination in large-scale collaborations and help young researchers to be faster introduced to best practices, tools and methods.

## Data Availability

The original contributions presented in the study are included in the article/supplementary material, further inquiries can be directed to the corresponding authors.
